# Transcriptome Sequencing of Zhikong Scallop (*Chlamys farreri*) and Comparative Transcriptomic Analysis with Yesso Scallop (*Patinopecten yessoensis*)

**DOI:** 10.1371/journal.pone.0063927

**Published:** 2013-05-07

**Authors:** Shan Wang, Rui Hou, Zhenmin Bao, Huixia Du, Yan He, Hailin Su, Yueyue Zhang, Xiaoteng Fu, Wenqian Jiao, Yan Li, Lingling Zhang, Shi Wang, Xiaoli Hu

**Affiliations:** Key Laboratory of Marine Genetics and Breeding (MGB), Ministry of Education, College of Marine Life Sciences, Ocean University of China, Qingdao, China; Georg August University of Göttingen, Germany

## Abstract

**Background:**

Bivalves play an important role in the ecosystems they inhabit and represent an important food source all over the world. So far limited genetic research has focused on this group of animals largely due to the lack of sufficient genetic or genomic resources. Here, we performed *de novo* transcriptome sequencing to produce the most comprehensive expressed sequence tag resource for Zhikong scallop (*Chlamys farreri*), and conducted the first transcriptome comparison for scallops.

**Results:**

In a single 454 sequencing run, 1,033,636 reads were produced and then assembled into 26,165 contigs. These contigs were then clustered into 24,437 isotigs and further grouped into 20,056 isogroups. About 47% of the isogroups showed significant matches to known proteins based on sequence similarity. Transcripts putatively involved in growth, reproduction and stress/immune-response were identified through Gene ontology (GO) and KEGG pathway analyses. Transcriptome comparison with Yesso scallop (*Patinopecten yessoensis*) revealed similar patterns of GO representation. Moreover, 38 putative fast-evolving genes were identified through analyzing the orthologous gene pairs between the two scallop species. More than 46,000 single nucleotide polymorphisms (SNPs) and 350 simple sequence repeats (SSRs) were also detected.

**Conclusion:**

Our study provides the most comprehensive transcriptomic resource currently available for *C. farreri*. Based on this resource, we performed the first large-scale transcriptome comparison between the two scallop species, *C. farreri* and *P. yessoensis*, and identified a number of putative fast-evolving genes, which may play an important role in scallop speciation and/or local adaptation. A large set of single nucleotide polymorphisms and simple sequence repeats were identified, which are ready for downstream marker development. This transcriptomic resource should lay an important foundation for future genetic or genomic studies on *C. farreri*.

## Introduction

Bivalves represent one of the oldest and evolutionarily most successful classes of invertebrates. They comprise 30,000 extant species which can adapt to a variety of marine and freshwater environments, although the molecular basis underlying these adaptations is still poorly understood. Bivalves play an important role in the ecosystems they inhabit [1]. For example, they act as extensive contributors to the transfer of mineral (e.g. calcium) and organic matter in benthic habitats. Moreover, bivalves serve as an important food source all over the world. In contrast with their ecological and economic significance, relatively less research attention has been paid to these animals. Moreover, most of bivalve studies carried out so far are biased towards a few well-studied species such as oysters and mussels. Obviously, future research on a broader range of bivalve species is very much encouraged to make a better understanding of bivalve adaptation and speciation.

The Pectinidae family, also known as scallops, consists of more than 300 extant species [2] and constitutes one of the most conspicuous groups of bivalves [3]. Among approximately 40 scallop species distributed along the coast of China, Zhikong scallop (*Chlamys farreri*, Jones et Preston 1904) represents one of the most important shellfish cultured in the north of China. Genetic breeding programs have recently been initiated for genetic improvement of this scallop species, and much research effort has been devoted to identify genes or genetic loci responsible for economically important traits such as rapid growth and disease resistance. For example, a number of growth- and immune-related genes have been cloned and characterized [4]–[10], and several growth-related quantitative trait loci (QTL) have also been identified [11]. However, none of these studies carried out so far has reached to the systems biology level, which is largely due to the lack of sufficient genetic or genomic resources for this scallop species. For example, as of 08/26/2012, there are only 3716 expressed sequence tags (ESTs) publicly available in the GenBank database for *C. farreri*, which are clearly far from representing the whole transcriptome of *C. farreri*.

Fortunately, the recent advent of next-generation sequencing (NGS) technologies that enables rapid and cost-effective large-scale sequencing shows great potential for expanding EST databases for potentially any non-model organisms, thus paving the way for functional genomics on scallops. In comparison with other NGS platforms such as Solexa and SOLiD, 454 sequencing technology can produce much longer reads, and therefore has been favorably chosen for *de novo* transcriptome sequencing in some ecologically and economically important bivalve species such as mussels [12], [13], clams [14], [15] and pearl oysters [16], [17]. Aside from gene discovery, many studies have demonstrated that transcriptome sequencing also represents an efficient way to discover genetic variations, e.g. single nucleotide polymorphisms (SNPs) and simple sequence repeats (SSRs), and help locating adaptive genes that are under natural selection. Meanwhile, the SNPs and SSRs discovered from transcriptome sequences, could be further developed to gene-based markers which are useful genetic tools in the studies on population genetics, QTL mapping, and pedigree assignment, etc [18].

Recently, our group has released a large amount of transcriptomic data for Yesso scallop (*Patinopecten yessoensis*) [19], providing the first NGS-based large-scale transcriptome resource available for scallops. This resource is valuable not only for gene discovery and molecular marker mining, but also for comparative transcriptomic analysis. Currently, almost nothing is known about the genetic bases underlying scallop adaptation and speciation. *P. yessoensis* is phylogenetically close to *C. farreri* [20] but differs remarkably in morphology and thermal preference. Transcriptome comparison between *P. yessoensis* and *C. farreri* may provide new insights into the processes of scallop adaptation and speciation.

In this study, we performed *de novo* transcriptome sequencing of *C. farreri* using the 454 GS FLX platform. A library representing diverse life stages and adult tissues of *C. farreri* was sequenced to identify groups of genes involved in a broad range of biological processes. Approximately 20,000 genes were identified which can serve as an important basis for further gene expression profiling studies. In comparison with the *P. yessoensis* transcriptome, 38 putative fast-evolving genes were identified. In addition, a large number of SSRs and SNPs were detected and are ready for marker development.

## Materials and Methods

### Sample collection and RNA preparation

All the experiments on scallops were conducted following the institutional and national guidelines. Embryos (blastulae and gastrulae), larvae (trochophore and D-shaped larvae) and adults of *C. farreri* were collected from the hatchery of Xunshan Group Co., Ltd (Shandong, China) in 2008. In addition to the total soft tissues, adductor muscle, male and female gonads were also independently dissected from sex-matured adults. All the samples were flash frozen in liquid nitrogen and stored at −80°C until use.

Total RNA was separately extracted from each sample by following the protocol previously described in Hu et al. [21]. The quantity and quality of total RNA was analyzed using an Ultrospec™ 2100 *pro* UV/Visible Spectrophotometer (Amersham Biosciences, Uppsala, Sweden) and gel electrophoresis. Equal amount of RNA from blastulae, gastrulae, trochophore larvae and D-shaped larvae was mixed as the embryo and larval RNA pool. Another four RNA samples for cDNA libraries construction were the total soft tissues of adults, adductor muscle, female gonad and male gonad. The technical details of these cDNA libraries were summarized in Table 1, and similar information was also displayed for *P. yessoensis*, which was retrieved from our previous study [19].

**Table 1:** Summary of the *C. farreri* and *P. yessoensis* cDNA libraries used for 454 sequencing.

Species		Developmental stages/adult tissues	No. of individuals used for library construction	Normalization
*C*. *farreri*	Library 1	Blastulae, Gastrulae, Trochophore, D-shaped larvae	∼1,000 for each stage	Yes
	Library 2	Total soft tissues	30	Yes
	Library 3	Adductor muscle	30	No
	Library 4	Male gonad	30	No
	Library 5	Female gonad	30	No
*P*. *yessoensis*	Library 1	Blastulae, Gastrulae, Trochophore, D-shaped larvae	∼1,000 for each stage	Yes
	Library 2	Adductor muscle	40	No
	Library 3	digestive gland	40	No
	Library 4	Male gonad	40	No
	Library 5	Female gonad	40	No

### cDNA library construction and 454 sequencing

Five 454 libraries (Table 1) were prepared by following the protocol as described in Meyer et al. [22]. Library 1 and 2 were normalized using the Trimmer-Direct cDNA normalization kit (Evrogen, Moscow, Russia) to decrease the prevalence of abundant transcripts. Library 3∼5 were not normalized in order to facilitate the identification of candidate tissue-specific transcripts. Approximately, 5 µg of the mixed libraries was sequenced using the Roche Genome Sequencer FLX system (Roche, Basel, Switzerland). During the construction of each library, adaptors with a unique barcode (a 3-base sequence) were ligated to both end of the cDNAs to distinguish the sequencing reads from those of other libraries. The reads were subsequently assigned to their corresponding libraries using Perl script [23].

### Sequence analysis and assembly

The raw 454 reads were first pre-processed by trimming adaptors and eliminating very short sequences (less than 100 bp). The pre-processed sequences were then subject to assembling using the program Newbler v2.5 (Roche) (cDNA assembly mode). Default assembly parameters were used with the minimum overlap length of 40 bp and the minimum sequence identity of 90%. The assembly program Newbler could account for alternative splicing by creating a hierarchical assembly composed of contigs, isotigs, and isogroups. It has been shown that this program is more efficient in assembling 454 reads than other assembling programs [24]. For transcriptome comparison, the 454 transcriptome sequences of *P. yessoensis* were retrieved from the NCBI SRA database under the accession no. SRA027310. To make a fair and reliable comparison, the clean reads of *P. yessoensis* were reassembled and annotated by following the same procedure described for *C. farreri*.

### Functional annotation

In order to avoid redundant annotations, only the longest isotig from each isogroup was selected and compared against the Swiss-Prot database using BlastX with an E-value threshold of 1e-6. For those isotigs without significant matches, tBlastX search with an E-value threshold of 1e-6 was conducted against the Nt database for further annotation. To increase computational speed, all Blast searches were limited to the top 10 significant hits for each query. Gene names were assigned to each isotig based on the best BLAST hit (highest score). The top 10 hits extracted from the BlastX results were used for gene annotation and GO analysis (level 3) using the program Blast2GO [25]–[27], a software package that assigned GO terms to query sequences, and produced a broad overview of groups of genes in the transcriptome cataloged for each of the three ontology vocabularies, i.e., biological processes, molecular functions and cellular components. The data presented herein represent a GO analysis at level 3, illustrating general functional categories.

In addition, to obtain an overview of gene pathways networks, KEGG analysis was performed using the online KEGG Automatic Annotation Server (KAAS) (http://www.genome.jp/kegg/kaas/). The bi-directional best hit (BBH) method was used to obtain KEGG orthology assignments.

### Ka/Ks analysis based on the putative orthologous sequences

The identification of putative orthologous ESTs between *C. farreri* and *P. yessoensis* was performed using the bidirectional best hit (BBH) approach [28]. Pairs of putative orthologous genes were identified based on the reciprocal best matches with an E-value threshold of 1e-6. To reduce the risk of comparing paralogs, we only retained those orthologous pairs by requiring both genes in each pair must show the best matches to the same protein when comparing against the SwissProt database (BlastX, E-value<1e-6) [29]. Since multi-gene families can confound the analysis, we required that for each orthologous gene pair, the annotated gene name must appear only once across all annotated isogroups within each transcriptome; otherwise, it will be excluded from further analysis. Coding sequences (CDSs) of the filtered orthologous gene pairs were determined from the BlastX results. CDSs with unexpected stop codons were removed. Ka (non-synonymous) and Ks (synonymous) values were calculated based on the orthologous CDSs using KaKs_Calculator [30]. Pair-wise approximate analyses were performed using the Yang and Nielsen method [31]. GO enrichment analysis was conducted through hypergeometric test for orthologous pairs that showed Ka/Ks values significantly deviated from 1, in order to find functionally coherent gene-sets that are statistically over-represented.

### SNP and SSR discovery

Potential SNPs were detected using the program GS Reference Mapper v2.6 with default parameters (cDNA mode). SNP identification was limited to the contigs containing at least eight reads for each allele and required the minor allele frequency ≥ 25%. SciRoko program version 3.3 [32] was used to identify and localize microsatellite motifs. All types of SSRs from dinucleotides to hexanucleotides were searched using default settings (for all repeat types, minimum total length = 15 bp and minimum repeats = 3).

## Results and Discussion

### Sequencing and assembly

The cDNA libraries representing different developmental stages, including embryos and larvae, and adults tissues of *C. farreri* were constructed and then pooled for 454 sequencing. The normalization and pooling strategies were used to enrich mRNA transcripts with low abundance and to maximize gene representation in a broad range of developmental and cellular processes. In addition, to obtain unique genes related to growth and reproduction which are both important economic traits for scallop, non-normalized cDNA libraries for adductor muscle, female gonad and male gonad were prepared separately (Table 1). A single run of 454 sequencing generated 1,224,989 reads. After removal of polyA tails, adaptor sequences and small reads (<100 bp), 1,033,636 (84.4%) high-quality reads remained with an average length of 310 bases. These high-quality reads have been deposited in the NCBI Short Read Archive (SRA) database with the accession number SRA030509. An overview of the sequencing and assembly statistics is presented in Table 2.

**Table 2:** Summary statistics of the transcriptome assembly for *C. farreri and P. yessoensis.*

	*C. farreri*	*P. yessoensis.*
Raw reads	1,224,989	970,422
Clean reads	1,033,636	740,491
Assembled reads	865,128	612,549
Contigs	26,165	13,306
Contig size N50	848 bp	898 bp
Average length of contigs	646 bp	688 bp
Mean no. of reads per contig	32	45
Isotigs	24,437	12,015
Isotig size N50	1,062 bp	1,121 bp
Average length of isotigs	868 bp	933 bp
Mean no. of contigs per isotig	1.4	1.6
Isogroups	20,056	10,147
Mean no. isotigs per isogroup	1.2	1.2

**Figure 1:**
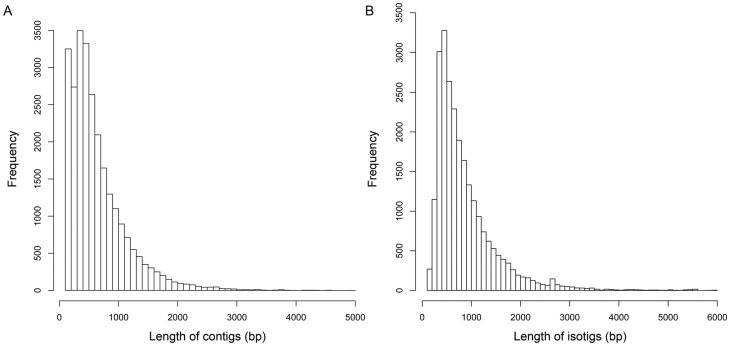
Overview of the *de novo* assembly of the *C.* *farreri* transcriptome. (A) Size distribution of contigs. (B) Size distribution of isotigs. Assembly of the high-quality reads produced 26,165 contigs with an average length of 646 bp (N50 = 848 bp). Contigs were further assembled into 24,437 isotigs with an average length of 868 bp (N50 = 1,062 bp).

Assembly of the high-quality reads produced 26,165 contigs with an average length of 646 bp (N50 = 848 bp). Approximately 84% of the high-quality reads were incorporated into these contigs. The average coverage of contigs was 32. Size distribution of these contigs is shown in Fig. 1A. More than 63% of the contigs were >500 bp. Contigs were then assembled into 24,437 isotigs with an average length of 868 bp (N50 = 1,062 bp). The size distribution of isotigs is shown in Fig. 1B. The average contig coverage for each isotig was 1.4. About 28.3% of the isotigs were >1,000 bp. The isotigs were further grouped into 20,056 isogroups. The remaining 168,506 reads that did not overlap with other sequences were considered as singletons. Although many singletons could represent useful lowly expressed transcripts, it is also possible that some are artifacts derived from cDNA synthesis, sequencing and contamination [22]. PCR validation or re-sequencing is necessary to verify the validity of these singletons. Hence these singletons were excluded from the following analyses.

### Functional annotation

Functional annotation (Table S1) of the *C. farreri* transcriptome was first carried out by the BlastX search against the well-annotated Swiss-Prot database with an E-value cut-off of 1e-6. Of 20,056 isogroup, 7,830 (39.0%) had significant matches in total, corresponding to 6,736 unique accessions. Among these accessions, 763 were matched by 1,857 different queries without overlap (2.4 queries matched each subject, on average). Sequences that lacked matches were subsequently compared against the Nt database (tBlastX) for further identification, and 1,498 additional isotigs returned a significant hit (E-value<1e-6). A large portion of the *C. farreri* transcriptome (53.5% of the isogroups) had no annotation information. The poor annotation efficiency was comparable to those reported in other *de novo* transcriptome sequencing studies based on the NGS platforms [22], [33]–[36]. This could be largely due to the insufficient sequences in public databases from phylogenetically closely related species to date [18]. Some of these sequences might represent novel proteins, unique to scallops, fast evolving genes or untranslated regions as well.

**Figure 2:**
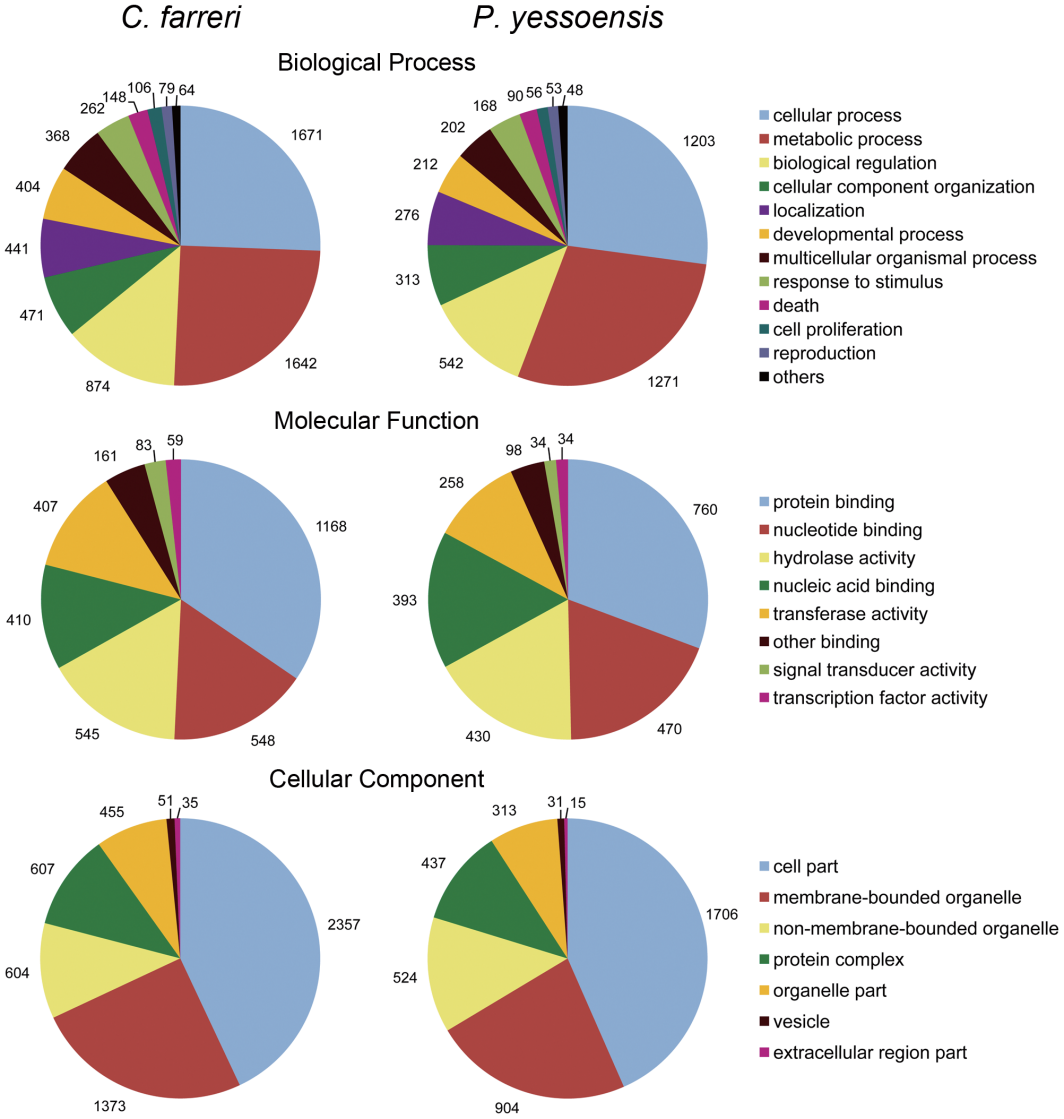
GO comparison between the *C.* *farreri* and *P. yessoensis* transcriptome. GO analysis was performed at the level 3 for three main categories (cellular component, molecular function and biological process). Note, for the two species, the starting materials used for cDNA library preparation and their normalization histories largely resemble each other, though not completely equivalent (see Table 1 for the details of libraries in comparison).

To classify the *C. farreri* genes based on their putative function, gene ontology (GO) analysis [37] was performed. Of the 7,830 Swiss-Prot annotated isotigs, 5,955 (76.1%) were assigned to one or more GO terms, with a total of 27,081 GO assignments. On average, about 5 GO terms were assigned to each of the annotated isotigs. Genes involved in the binding (GO:0005488) and catalytic activity categories (GO:0003824) were highly represented in molecular function. Within cellular component, the most represented GO categories were cell (GO:0005623) and organelle (GO:0043226). Regarding biological process, cellular process (GO:0009987) was the most represented, followed by metabolic process (GO:0008152) (Fig. 2).

As an alternative approach of categorizing the annotated isogroups based on biochemical pathways, KEGG analysis based on enzyme commission (EC) numbers was performed for all annotated sequences using the KEGG Automatic Annotation Server (KAAS) [38]. EC numbers were assigned to 2,062 isogroups which were involved in 229 different pathways. The isogroups involved in these pathways are summarized in Table 3. Of these 2,062 isogroups with KEGG annotation, 38.7% were classified into the genetic information processing pathways, with most of them involved in translation, folding, sorting and degradation, and transcription. The isogroups classified into metabolism accounted for 33.9% of the KEGG annotated sequences. The well-represented metabolic pathways were amino acid metabolism, carbohydrate metabolism, and lipid metabolism. About 22.7% of the isogroups were classified into organism systems, such as immune system, endocrine system, and nervous system. Cellular processes were represented by 22.6% of the KEGG annotated isogroups. The transport and catabolism, cell growth and death, and cell communication were well represented. Additionally, 13.3% of the isogroups involved environmental information processing, including signal transduction, signaling molecules and interaction, and membrane transport.

**Table 3:** KEGG biochemical mappings for *C. farreri* transcriptome.

KEGG categories represented	Unique sequences (Number of enzymes)
**Metabolism**	**698 (572)**
Amino Acid Metabolism	146 (123)
Carbohydrate Metabolism	134 (107)
Lipid Metabolism	134 (108)
Energy Metabolism	124 (112)
Nucleotide Metabolism	102 (83)
Glycan Biosynthesis and Metabolism	77 (64)
Metabolism of Cofactors and Vitamins	72 (61)
Metabolism of Other Amino Acids	64 (45)
Xenobiotics Biodegradation and Metabolism	63 (41)
Metabolism of Terpenoids and Polyketides	15 (13)
Biosynthesis of Other Secondary Metabolites	13 (12)
**Genetic Information Processing**	**799 (692)**
Translation Replication and Repair	303 (263)
Folding, Sorting and Degradation	276 (232)
Transcription	162 (136)
Replication and Repair	99 (86)
**Environmental Information Processing**	**275 (228)**
Signal Transduction	215 (179)
Signaling Molecules and Interaction	61 (50)
Membrane Transport	10 (8)
**Cellular Processes**	**465 (390)**
Transport and Catabolism	224 (173)
Cell Growth and Death	152 (114)
Cell Communication	106 (86)
Cell Motility	50 (43)
**Organismal Systems**	**469 (390)**
Immune System	158 (128)
Endocrine System	137 (114)
Nervous System	133 (109)
Digestive System	93 (71)
Development	63 (54)
Excretory System	54 (47)
Circulatory System	46 (36)
Environmental Adaptation	21 (18)
Sensory System	18 (13)
**Total**	**2,062 (1,726)**

For scallops, growth and reproduction are economically important traits, thus genes involved in these processes are of particular interest to the scallop researchers for the purpose of genetic improvement. Genes encoding different groups of growth factors, such as EGF, TGF, IGF and FGF, as well as their receptors were identified. Regarding reproduction, genes encoding DEAD-box family members (e.g. *vasa*, *PL10* and *eIF4A*) that are involved in the germ cell development and reproductive regulation [39]–[41], and Piwi-like proteins that are responsible for maintaining the stability of germline cell division rate were identified [42]. The cDNAs from adductor muscle and gonad were not experimentally normalized, so as to assess the transcripts putatively related to the function of these tissues. In adductor muscle, structural or muscle-related genes such as *actin*, *myosin*, *tubulin*, and *troponin* were highly expressed. Transcripts of *myostatin* which were proved to be an important regulator of muscle growth and development in vertebrates were only found in the cDNAs from scallop adductor muscle. In male and female gonad, the most highly expressed gene was sperm-specific H1/protamine-like protein and collagen-like protein, respectively. Other putative sex-specific transcripts encoding sperm-specific proteins, vitellogenins and estradiol dehydrogenase, *etc*. [43]–[45], were also discovered. Further GO analysis also identified sequences classified into terms associated with growth and reproduction (Table 4).

**Table 4:** Sequences classified into growth, reproduction and response to stimulus categories by GO analysis.

GO terms	Number of sequences
**growth (GO:0040007)**	**47**
cell growth (GO:0016049)	25
regulation of growth (GO:0040008)	5
multicellular organism growth (GO:0035264)	2
negative regulation of growth (GO:0045926)	1
**reproduction (GO:0000003)**	**79**
**response to stimulus (response to stimulus)**	**262**
response to stress (GO:0006950)	211
response to external stimulus (GO:0009605)	48
behavior (GO:0007610)	36
response to abiotic stimulus (GO:0009628)	33
response to biotic stimulus (GO:0009607)	27
response to endogenous stimulus (GO:0009719)	27
immune response (GO:0006955)	22

With the increasing environmental pressure on natural and farmed scallop populations largely resulting from the increasing use of coastal zones, research efforts have recently been devoted to understanding of the genetic bases of stress-resistance in scallops. In our study, both GO and KEGG analysis identified transcripts that are involved in cellular responses to environmental pressure and stimulus (Table 4 and Table 5). The GO analysis identified 198 and 262 transcripts that are related to stress responses (GO: 0006950) and stimulus responses (GO: 0050896), respectively. KEGG analysis showed that 13.3% of the isogroups belonged to environmental information processing (EIP), including signal transduction, signaling molecules and interaction, and membrane transport. In addition, 7.7% of the isogroups were involved in immune response. Further functional analysis of these genes may provide valuable information for understanding of the genetic basis underlying scallop stress-resistance and productive traits.

**Table 5:** Sequences classified into Immune System by KEGG analysis.

KEGG Pathways	Number of sequences
Hematopoietic cell lineage	7
Complement and coagulation cascades	9
Toll-like receptor signaling pathway	23
NOD-like receptor signaling pathway	13
RIG-I-like receptor signaling pathway	18
Cytosolic DNA-sensing pathway	21
Natural killer cell mediated cytotoxicity	21
Antigen processing and presentation	16
T cell receptor signaling pathway	22
B cell receptor signaling pathway	22
Fc epsilon RI signaling pathway	16
Fc gamma R-mediated phagocytosis	27
Leukocyte transendothelial migration	21
Chemokine signaling pathway	31
**Total**	**267**

### Transcriptome comparison between *C. farreri* and *P. yessoensis*

Many studies have demonstrated the usefulness of next-generation sequencing in obtaining transcriptomic resources for comparative analysis in non-model organisms [46], [47]. Here, for the first time, we performed scallop transcriptome comparison by comparative analysis of the new EST data set generated for *C. farreri* with the one recently published for *P. yessoensis* [19]. To make a fair and reliable comparison, the sequencing data of *P. yessoensis* transcriptome was reassembled and annotated by following the same procedure as described for *C. farreri*. The assembled transcriptome of *C. farreri* and *P. yessoensis* contained 20,056 and 10,147 isogroups, respectively. For the two species, the starting materials used for cDNA library preparation and their normalization histories largely resemble each other, though not completely equivalent (Table 1). For example, a normalized cDNA library of total soft tissues was sequenced for *C. farreri*, but not for *P. yessoensis*. In addition, it is possible that not all transcripts have been adequately sampled from all tissues and developmental stages based on the current sequencing coverage. Despite these discrepancies, transcriptome comparison between *C. farreri* and *P. yessoensis* based on the available EST sequences remains worth doing and should represent the first step towards full understanding of transcriptome organization and evolution in the Pectinidae family.

To get an overall comparison of the transcriptome organization between the two scallop species, we first analyzed their transcriptome sequences in terms of functional annotation and relative abundance of gene ontology (GO) terms. A total of 5,955 *C. farreri* isogroups and 3,533 *P. yessoensis* isogroups were assigned to 27,081 and 16,682 GO terms, respectively. Similar transcriptome pattern in terms of GO categories and their relative frequencies were found between *C. farreri* and *P. yessoensis* (Fig. 2), suggesting the overall similar transcriptome architecture between the two species. Further analyses with batches of cDNA sequences from more tissues and embryo/larva at more developmental stages for both *C. farreri* and *P. yessoensis* are needed to compare their transcripomes more thoroughly.

To enable Ka/Ks analysis, putative orthologous gene pairs were determined through a series of stringent filtering steps. Through the BBH approach, an initial set of 5,367 putative orthologous pairs were first identified. This number was reduced to 2,847 by requiring both genes in each pair must show the best matches to the same protein when comparing against the SwissProt database (BlastX, E-value<1e-6). Since multi-gene families can confound the analysis, we further removed the orthologous pairs whose gene names appeared more than once across all annotated isogroups within each transcriptome. The final set consisted of 1,887 pairs, which were selected for further analysis.

Most of the orthologous pairs (1,709) showed a Ka/Ks ratio<1. Among these, 1,644 orthologous pairs had Ka/Ks ratios significantly<1 (Table S2). Many sequences had Ka/Ks values equal to or only slightly greater than zero, suggesting that these genes have evolved under high selective constraint [48]. On the other hand, 178 orthologous pairs had Ka/Ks ratios >1. And 38 genes, which exhibited Ka/Ks values significantly deviated from 1 (Table S2), are possibly under positive selection and may play important roles in scallop adaptation and speciation. GO enrichment analysis (p<0.05) was performed for orthologous pairs to find functionally coherent gene-sets that are statistically over-represented. Genes with Ka/Ks values significantly<1 were enriched in a variety of biological processes such as metabolic process (p = 9.6E-14), translation (p = 1.3E-09) and biosynthetic process (p = 5.8E-07) (Table S3). However, genes with Ka/Ks values significantly >1 were only enriched in cellular biosynthetic process (p = 4.6E-04) and gene expression (p = 4.8E-04) (Table S3), suggesting that these terms probably represent the most important biological processes responsible for rapid adaptive evolution.

### SNP and SSR discovery

For genetic improvement of *C. farreri*, a large number of molecular markers are usually required for fine QTL mapping and marker-assisted selection (MAS). The transcriptome data here provided a rich EST resource for genetic variants mining, such as SNP and SSR screening.

**Figure 3:**
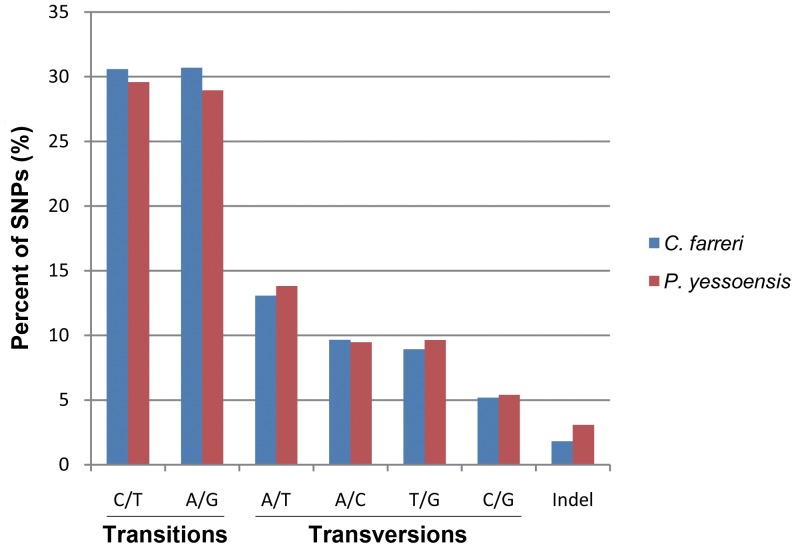
Classification of single nucleotide polymorphisms (SNPs) identified from the *C.* *farreri* and *P. yessoensis* transcriptome. For both species, transitions occurred more frequently than transversions. The overall frequency of all types of SNPs including indels was one per 357 bp for *C. farreri* and one per 430 bp for *P. yessoensis*.

Using the GS Reference Mapper program, we were able to identify 46,527 high-quality SNPs and 866 indels from 9,989 contigs. The overall frequency of all types of SNPs including indels was one per 357 bp. To make a comparison, SNPs in the reassembled *P. yessoensis* transcriptome were also examined. As a result, 20,633 SNPs and 658 indels were identified. The frequency of SNPs and indels was one per 430 bp. In the two species, the distribution of each SNP type was similar, and transitions occurred more frequently than transversions (Fig. 3). The proportion of transitions in *C. farreri* was higher than that in *P. yessoensis*, while the transversions were more abundant in *P. yessoensis*. A/T was the most abundant transversions type and C/G was the least in both species. Indels occurred the least frequently compared with transitions and transversions in the two species. Unlike other SNP types which showed not much difference in proportion between the two scallops, the indels proportion in *P. yessoensis* was 1.7 times as much as that in *C. farreri*.

In addition, 352 and 213 SSRs were identified from the transcriptome sequences of *C. farreri* and *P. yessoensis*, respectively. Comparison between the two scallop species revealed some difference in distribution pattern of SSR motifs (Table 6B). Trinucleotide and tetranucleotide were the most frequent type of repeats in both *C. farreri* and *P. yessoensis*. Although dinucleotide repeats were the third most frequent type in *C. farreri*, they were the least abundant type in *P. yessoensis*. ATC motif represented the most abundant trinucleotide motif in both species, which was also common in other bivalves [49]. For dinucleotide and pentanucleotide repeats, AT and AAAAT was the most frequent motif in the two species. But for tetranucleotides, the most frequent motif in *C. farreri* was ATAC, while in *P. yessoensis* was AAAT. A notable difference also existed in hexanucleotides. AAGGTC was the most common hexanucleotide motif in *C. farreri*, but only one copy was found in *P. yessoensis*.

**Table 6:** Summary of simple sequence repeat (SSR) types in C. farreri and *P. yessoensis* transcriptome.

	*C. farreri*			*P. yessoensis*		
SSR Type	Number of motif	Count	Major motif	Number of motif	Count	Major motif
Dinucleotides	3	50	AT	3	24	AT
Trinucleotides	9	164	ATC	8	104	ATC
Tetranucleotides	16	60	ATAC	14	31	AAAT
Pentanucleotides	18	46	AAAAT	18	29	AAAAT
Hexanucleotides	22	32	AAGGTC	19	25	AACTGG

The SNPs and SSRs identified in this study provided for the first time over thousands of putative candidate loci for marker development in *C. farreri*. In case that there might be sequencing errors or assembly artifacts, the bioinformatically discovered SNPs and SSRs should be further validated and evaluated for marker utility in the *C. farreri* natural populations. A small portion of the SNPs identified in this study have already been assessed in two separate studies, and some of them were successfully developed as gene-associated polymorphic markers [50], [51], which could be very useful in future selective breeding and population genetic studies for *C. farreri*.

## Supporting Information

Table S1 **Sequences with significant BLAST matches against Swiss-Prot and NCBI Nt database.**
(XLS)

Table S2 **Putative orthologous genes with Ka/Ks values significantly different from one (p<0.05).**
(XLS)

Table S3 **GO enrichment analysis for putative orthologous genes with Ka/Ks values significantly different from one.**
(DOC)
