# Supplementary material for: Transcriptome Sequencing of Zhikong Scallop (Chlamys farreri) and Comparative Transcriptomic Analysis with Yesso Scallop (Patinopecten yessoensis)
Source: PLoS One. 2013 May 7;8(5):e63927. doi: 10.1371/journal.pone.0063927 (PMC3646770; doi:10.1371/journal.pone.0063927)
Supplement: Table S3 — GO enrichment analysis for putative orthologous genes with Ka/Ks values significantly different from one. (DOC) [file pone.0063927.s003.doc]

**Table S3.** **GO enrichment analysis for putative orthologous genes with Ka/Ks values significantly different from one.**

|  | GO ID | Term | N1 | N2 | p-value |
| --- | --- | --- | --- | --- | --- |
| Ka/Ks>1 | GO:0044249 | cellular biosynthetic process | 466 | 11 | 4.60E-04 |
|  | GO:0010467 | gene expression | 468 | 11 | 4.80E-04 |
| Ka/Ks<1 | GO:0008152 | metabolic process | 1642 | 482 | 9.60E-14 |
|  | GO:0009987 | cellular process | 1671 | 460 | 2.10E-06 |
|  | GO:0044237 | cellular metabolic process | 1166 | 368 | 1.20E-13 |
|  | GO:0044238 | primary metabolic process | 1438 | 429 | 4.00E-12 |
|  | GO:0009058 | biosynthetic process | 727 | 228 | 5.80E-07 |
|  | GO:0043170 | macromolecule metabolic process | 929 | 279 | 1.30E-06 |
|  | GO:0006807 | nitrogen compound metabolic process | 712 | 220 | 4.00E-06 |
|  | GO:0009056 | catabolic process | 297 | 102 | 3.80E-05 |
|  | GO:0019538 | protein metabolic process | 573 | 189 | 1.60E-07 |
|  | GO:0006091 | generation of precursor metabolites and energy | 69 | 35 | 1.80E-06 |
|  | GO:0006139 | nucleobase, nucleoside, nucleotide and nucleic acid metabolic process | 712 | 220 | 4.00E-06 |
|  | GO:0044249 | cellular biosynthetic process | 466 | 151 | 1.50E-05 |
|  | GO:0009059 | macromolecule biosynthetic process | 466 | 151 | 1.50E-05 |
|  | GO:0010467 | gene expression | 468 | 151 | 2.00E-05 |
|  | GO:0044260 | cellular macromolecule metabolic process | 782 | 231 | 8.80E-05 |
|  | GO:0044267 | cellular protein metabolic process | 410 | 137 | 6.90E-06 |
|  | GO:0034645 | cellular macromolecule biosynthetic process | 466 | 151 | 1.50E-05 |
|  | GO:0006412 | translation | 136 | 65 | 1.30E-09 |

N1, Number of genes from *C. farreri* transcriptome in the given term.

N2, Number of putative orthologous gene pairs with Ka/Ks values significantly higher or lower than one in the given term.

All the terms listed were signiﬁcantly enriched after Bonferroni correction (p < 0.05).
